# Advisory groups in realist reviews: Systematically mapping current research and recommendations for practice

**DOI:** 10.1002/cesm.12073

**Published:** 2024-06-11

**Authors:** Jessica Power, Sara Dada, Andrew Booth, Aoife De Brún, Brynne Gilmore

**Affiliations:** ^1^ School of Medicine, Trinity College Dublin Dublin Ireland; ^2^ UCD Centre for Interdisciplinary Research Education and Innovation in Health Systems (UCD IRIS Centre), School of Nursing Midwifery and Health Systems University College Dublin Dublin Ireland; ^3^ School of Health and Related Research (ScHARR) University of Sheffield Sheffield UK; ^4^ University of Limerick Limerick Ireland

**Keywords:** advisory groups, realist review, realist synthesis, stakeholder involvement

## Abstract

**Introduction:**

Realist reviews may involve groups or panels external to the research team who provide external and independent perspectives informing the review based on their experience of the topic area. These panels or groups are termed in this study as an “advisory group.” This study aims to map current practice of advisory groups in realist reviews and provide guidance for planning and reporting.

**Methods:**

A “best‐fit” framework synthesis methodology was used by first searching for a best‐fit framework and then conducting a systematic search to identify a sample of realist reviews and rapid realist reviews (RRRs) from the most recent year, 2021. Nine databases were searched: CINAHL Complete, Cochrane, Embase, ERIC, MEDLINE, PsycInfo, Social Services Abstracts, Sociological Abstracts, and Web of Science Core Collection. Screening and data extraction was conducted by two researchers. The chosen best‐fit framework (ACTIVE framework) informed the data extraction tool.

**Results:**

One hundred and seven reviews (93 realist reviews, 14 RRRs) were identified for inclusion. Of these, 40% (*n* = 37) of realist reviews and 71.5% (*n* = 10) of RRRs mentioned use of an advisory group, though there was considerable variation in terminology used. Individuals in advisory groups were involved at varying stages of the review and tended to bring experience in the topic area from the perspective of (i) a lived experience, i.e., patients, carers, family members (*n* = 15 realist reviews; *n* = 4 RRRs); (ii) professional experience, such as healthcare professionals (*n* = 20 realist reviews; *n* = 6 RRRs); or (iii) policy or research experience in the topic area (*n* = 19 realist reviews; *n* = 7 RRRs).

**Conclusions:**

This study proposes a definition of advisory groups, considerations for advisory group use, and suggested items for reporting. The purpose of the advisory group should be carefully considered when deciding on their use in a realist review.

## 1 INTRODUCTION

Realist review (also known as realist synthesis) is a theory‐driven form of evidence synthesis [1]. Review authors typically seek to explain how and why an intervention may work (or not) across different settings [2]. This form of evidence synthesis differs from conventional systematic reviews in numerous ways, including its iterative approach involving multiple searches in response to emergent needs of the review [1, 3]. Realist reviews may use an external group or panel to support and inform the iterative review process. Different terminology is used to describe these groups, for example, “advisory groups,” “expert groups,” “expert panels,” or “stakeholder groups.” For the purpose of this study, we will use the term advisory groups, to encompass where a group or panel external to the research team was used to inform and guide the review.

Realist reviews can be categorized into standard realist reviews and “rapid” realist reviews (RRRs) [4]. RRRs may be completed in a shorter timeline and may place more emphasis and guidance on engaging with knowledge users and experts to input into the review through a local reference group and expert panel [4]. The use of an advisory group and/or content experts, although mentioned in guidance materials [1] and quality standards [5] for realist reviews, has attracted only minimal information or guidance on its use. For standard realist reviews, the use of stakeholder input is discussed primarily in relation to focusing the review question and in developing and refining initial theory. Therefore, “standard” realist reviews, referred from here onwards as realist reviews, receive little information on what an advisory group is or what is best practice for their use. While guidance materials were not intended to be prescriptive, offering flexibility to the needs and available resources of each review [1], it does leave those carrying out realist reviews with little direction on what an advisory group is and how they can be leveraged/harnessed to support a review best.

Stakeholder involvement in other health services research [6, 7] and other review types such as scoping [8] and systematic reviews [9, 10, 11] has been previously studied. Abrams et al. [12] explored the involvement of stakeholders, patients, public and nonresearch contributors in realist reviews, specifically focusing on patient and public involvement (PPI). They noted a lack of clarity of terminology as well as a lack in reporting who was involved and the impact of their involvement on the review in most of the studies identified. While they reported that PPI could include being part of an advisory group, their study did not specifically seek to define what an advisory group is in a realist review. Additionally, while advisory groups may have public and patient involvement, this may not always be the case with some groups solely having professionals as members [4]. Abrams et al. [12] identified the lack of guidance for external involvement in realist reviews, suggesting that existing frameworks in other forms of evidence synthesis could be used to inform guidance for realist reviews as well. This study aims to build upon this work by focussing specifically on advisory groups in realist reviews, to provide a definition of advisory groups, and to build upon existing frameworks in evidence synthesis to support future realist review studies using advisory groups.

As realist reviews gain popularity, their methods are evolving with much learning to be captured from current practice [3, 13]. Within realist reviews, advisory groups have taken place in varying degrees and at various stages in the review [3, 12]. For example, advisory groups have been used when focusing the review question [14], contributing to the search strategy [15], developing initial theories [16], and refining and validating theory [16, 17]. However there is a lack of clarity around what an advisory group is, how they are used, and what should be reported. The present study aims to collate and learn from current practice in realist reviews to define what is an advisory group in a realist review, how they are used in practice, and why they are used. Based on this learning, this study aims to provide a framework with recommendations for consideration to guide the use of advisory groups for future realist reviews/syntheses together with suggestions for reporting.

## 2 MATERIALS AND METHODS

This study occurred across two main stages using a “best fit” framework synthesis approach, adapted from Carroll et al. [18, 19] First, a search took place for a potential “best fit” a priori framework for the study. Second, a systematic search took place for a recent single year sample of realist reviews from which data relating to using an advisory group was extracted. Following this, an operational definition for what is an advisory group was developed, and the selected “best fit” framework was built upon to provide guidance for considering the use of an advisory group and suggested items for reporting.

We first sought to identify a “best fit” framework [18, 19] from existing frameworks of advisory group or stakeholder involvement in other evidence syntheses, such as systematic reviews and health services research. Five known frameworks were used as a starting point for the search [6, 9, 10, 11, 20]. For each of these frameworks, (i) reference list searches and (ii) forward citation searches in Google Scholar took place in September 2022 (see Supporting Information S1: File S1). Eight potential frameworks were shortlisted through this search [6, 8, 9, 11, 21, 22, 23, 24], which were discussed between two members of the team (Jessica Power and Brynne Gilmore). Following this, the ACTIVE framework [9], developed for stakeholder input in systematic reviews, was selected and taken to the wider research team for further discussion and agreement for use as an initial framework for the study.

Second, a systematic search took place to identify a sample of realist reviews published within the most recently completed year. The search strategy was adapted from Booth et al. [13] with search terms used for realist review/synthesis. The following databases were searched: CINAHL Complete, Cochrane, Embase, ERIC, MEDLINE, PsycInfo, Social Services Abstracts, Sociological Abstracts, and Web of Science Core Collection. This search took place in September 2022 (see Supporting Information S2: File S2).

Given the resources available, we decided to limit searches to a sample from the most recently completed single year of 2021. Reviews which named themselves as a realist review, and also followed RAMESES criteria [1, 5] for realist review/synthesis, were included. Reviews not using this approach or which were reported as a realist‐informed, critical realist, or another systematic review type, were excluded. Realist evaluations were excluded, however, publications jointly reporting both a realist review and evaluation were included, with data taken only from the realist review component. RRRs were included and were analyzed separately. Protocols of reviews in progress or not yet published were excluded; however, if a completed realist review referenced a previously published protocol for detail on the advisory group, this was considered as supporting information attributed to the index review. To note these studies were coded as using an advisory group if they described a group or panel external to the research team which was used to inform and guide the review. However, if they described stakeholders as an individual participant, for example, where interviews were transcribed and analyzed, this was not coded as an advisory group. Table 1 exhibits the inclusion and exclusion criteria. Two reviewers (Jessica Power and Sara Dada) screened records independently using Covidence, first by title/abstract and then by full text with reasons for exclusion recorded. A third reviewer (Brynne Gilmore) was available for any disagreements.

**Table 1:** Inclusion and exclusion criteria for search to identify sample of realist reviews.

Inclusion criteria Realist review/synthesis published in a peer‐reviewed journal in 2021 Realist review/synthesis as part of a larger study (e.g., realist review and evaluation) published in a peer‐reviewed journal in 2021 Rapid realist review published in a peer‐reviewed journal in 2021 English language
Exclusion criteria Realist review/synthesis published before or after 2021 Protocol only but no final review published Realist evaluation but did not include a review/synthesis Realist informed but does not fit RAMESES criteria for realist review/synthesis Conference abstracts, book chapters, thesis

The selected “best fit” framework [9] was used as a starting point for the data extraction tool, which was adapted and piloted by the research team. Extracted data included: the terminology used for the advisory group, stage(s) and manner of involvement in the review, recruitment approaches and membership of the advisory group (Supporting Information S1: File S3). Data extraction was completed by two reviewers (Jessica Power and Sara Dada) in Covidence. Initially, data from five review publications were extracted independently and compared between reviewers for consensus. Following this, a further five reviews were extracted and compared with a good level agreement. The remaining studies were divided between the two reviewers for extraction, however, reviews where advisory group participation was unclear were tagged and extracted by a second reviewer and agreement was reached on details for extraction. A third reviewer (Brynne Gilmore) conducted a quality check of 20% of the reviews. Once extraction was completed, data were exported to Excel for analysis.

## 3 RESULTS

**Figure 1:**
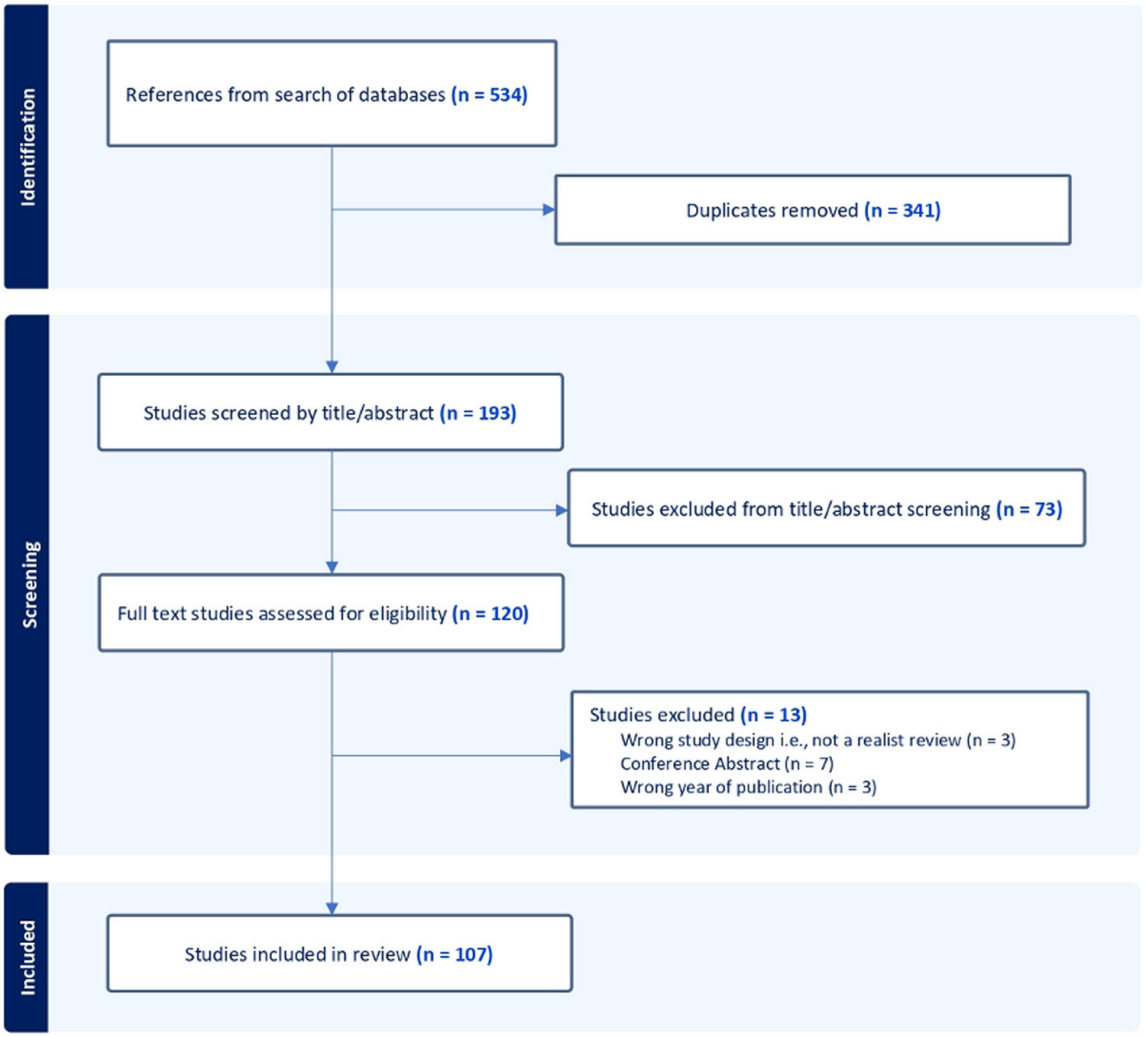
Flowchart outlining processing of screening for identifying a sample of realist reviews.

The systematic search yielded 537 results, with 198 remaining following the removal of duplicates. After title/abstract screening, 120 publications were screened at full text. A total of 107 reviews were identified for inclusion. Of these, 93 were realist reviews, and 14 were rapid realist reviews. Figure 1 provides a flowchart of the screening process and documents reasons for exclusion. 40% (*n* = 37) of the realist reviews cited use of an advisory group at some stage (as per the working definition for this study, notably other terminology was often reported) [14, 15, 16, 17, 25, 26, 27, 28, 29, 30, 31, 32, 33, 34, 35, 36, 37, 38, 39, 40, 41, 42, 43, 44, 45, 46, 47, 48, 49, 50, 51, 52, 53, 54, 55, 56, 57, 58, 59, 60, 61]. 60% (*n* = 56) did not mention the use of an advisory group [62, 63, 64, 65, 66, 67, 68, 69, 70, 71, 72, 73, 74, 75, 76, 77, 78, 79, 80, 81, 82, 83, 84, 85, 86, 87, 88, 89, 90, 91, 92, 93, 94, 95, 96, 97, 98, 99, 100, 101, 102, 103, 104, 105, 106, 107, 108, 109, 110, 111, 112, 113, 114, 115, 116, 117]. Of those that did not mention an advisory group, seven reviews [62, 63, 64] did report participant involvement that did not fulfill the criteria of an advisory group, i.e., participants were used as data sources but did not advise on the review. Advisory group use was more common in RRRs; 71.5% (*n* = 10) mentioning the use of an advisory group [14, 43, 44, 45, 118, 119, 120, 121, 122, 123], while the remaining 28.5% (*n* = 4) did not mention an advisory group [124, 125, 126, 127].

### 3.1 Who was involved in the advisory group?

Individuals involved in advisory groups tended to bring experience in the topic area from the perspective of (i) a lived experience, i.e., patients, carers, family members (*n* = 15 realist review; *n* = 4 RRRs); (ii) professional experience, such as healthcare professionals (*n* = 20 realist reviews; *n* = 6 RRRs); or (iii) policy or research experience in the topic area (*n* = 19 realist reviews; *n* = 7 RRRs). An advisory group could draw members from all the above categories, as illustrated by Duddy and Wong's [35] group of 14 stakeholders, which included patients, members of the public, clinicians, a laboratory scientist, and a policymaker. To a lesser extent, members with methodological experience in realist methods were reported (*n* = 3 realist reviews; *n* = 2 RRRs). Eight realist reviews and two RRRs did not report sufficient detail to identify who was involved

Some reviews also acknowledged that the research team had expertise in the topic of interest. For example, Kinsey et al. [125] reported that an advisory group was not used in their RRR, but the topic expertise of the review team contributed to IPT development. Another study named authors as expert panel members [118], however it was unclear at what point in the review process they became review team members and authors of the study.

### 3.2 How were advisory groups recruited?

Generally, minimal information was given on how advisory group members were recruited. For example, Wade et al. [29] explained their youth advisory group was formed by “advertising through batyr, a for‐purpose preventative mental health organization in Australia.” Those that did give information described sending invitations to existing PPI groups [26, 45] or using a pre‐established advisory group panel from a wider study, supplemented with additional stakeholders [57]. In contrast, one review [27] reported actively trying to recruit outside of established PPI groups to engage with new points of view.

### 3.3 Mode and frequency of involvement of advisory group

There was minimal reporting of how often or in what way the advisory groups met, with over half presenting no details on this (*n* = 20 realist review; *n* = 6 RRR). Those who provided details reported interacting on more than one occasion (*n* = 14 realist review; *n* = 4 RRR) or just once (*n* = 2 realist review). A face‐to‐face or online meeting was the most frequently reported mode of interaction (*n* = 11 realist review; *n* = 5 RRR), with some reviews reporting additional interaction to review documentation via email (*n* = 5 realist review; *n* = 1 RRR). For example, Sims et al. [33] reported that the group met three times and reviewed documents in between via email. Workshops (*n* = 4 realist review; *n* = 1 RRR) and interaction on the phone (*n* = 2 realist review) were also reported. To note that these studies were published in 2021 where COVID restrictions may have impacted the mode of interaction and may not represent usual practice. While the most common interaction was a meeting, it was noted that meetings were not always the most appropriate platform to engage the advisory group depending on the subject matter. For example, in their review of the remediation of medical doctors, Price et al. [32] felt it was more appropriate to liaise individually with advisory group members as opposed to a collective group meeting due to the sensitivity of the subject matter.

### 3.4 Stage of realist review

**Figure 2:**
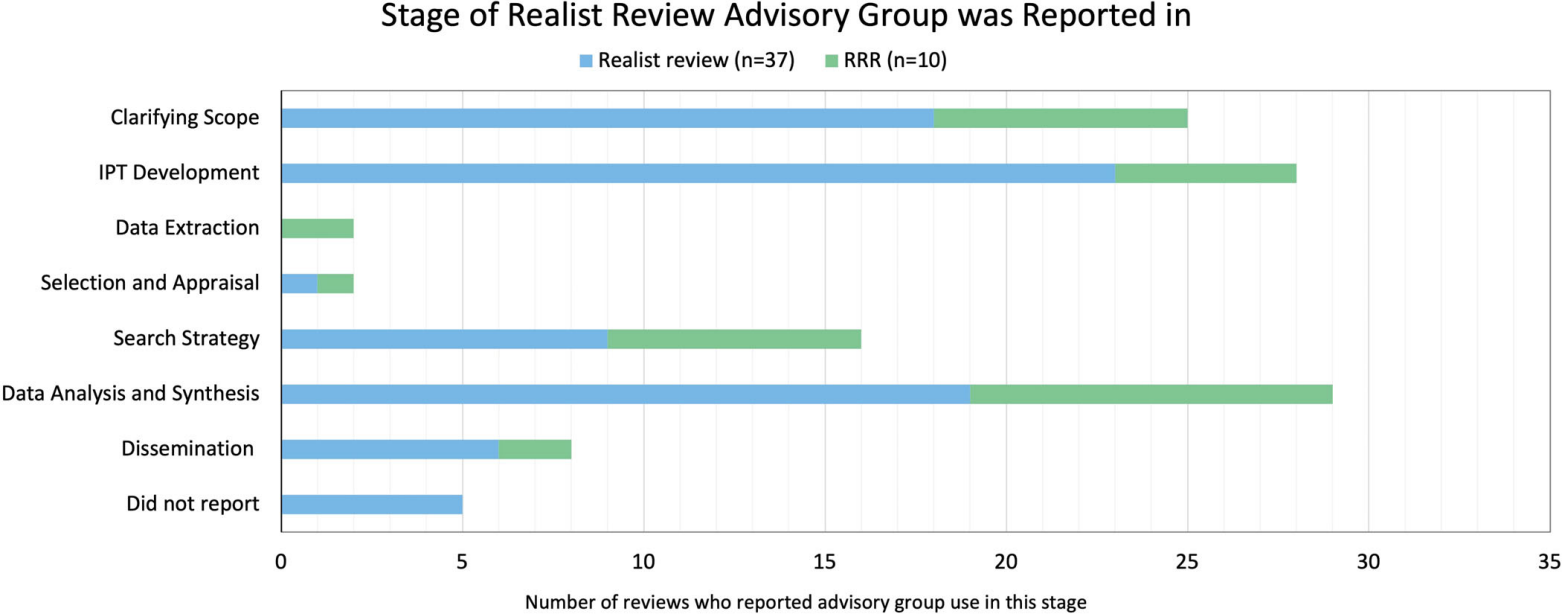
Stage of the realist review advisory group involvement was reported. To note, involvement in more than one stage is often reported.

Pawson et al. [128] outlined the key stages of the realist review as (i) clarifying the scope and developing initial rough theory, (ii) search strategy, (iii) selection and appraisal, (iv) data extraction, (v) data analysis and synthesis, including theory validation. These stages can be carried out iteratively as the needs of the review evolve [128]. Advisory groups were reportedly involved in consulting and advising at different stages of the realist review, often with involvement in more than one stage. Advisory group involvement was extracted separately for the stages “clarifying scope” and “initial program theory (IPT) development,” as their involvement was not always reported in both. Therefore these two separate headings were used instead of “clarifying the scope and developing initial rough theory.” Additionally, the dissemination step was included as it was reported as a distinct role in the included reviews. Figure 2 provides an overview of the stages of the realist review where advisory groups were involved. Table 2 lists the realist reviews and RRRs which used an advisory group and at what stage they used them.

**Table 2:** Overview of studies who mentioned an advisory group and stage of review advisory group involved in (where reported).

Review	Stage of Realist Review Advisory Group was reported to be involved in							
	Clarifying scope	IPT development	Search strategy	Selection and appraisal	Data extraction	Data analysis and synthesis	Dissemination	Not enough detail to code
Aunger et al. [40]		x						
Bansal et al. [36]	x	x						
Berkman et al. [41]	x	x					x	
Booth et al. [13]								
Burton et al. [52]	x	x				x	x	
Calderon‐Larranaga et al. [25]	x					x		
Carswell et al. [17]	x					x		
Chadborn et al. [16]		x	X			x		
Davidson et al. [48]	x							
Dixon et al. [30]		x	X			x		
Duddy and Wong [35]		x				x		
Dunn et al. [34]								x
Eaton et al. [56]	x					x		
Fernandez Turienzo et al. [61]	x					x		
Fowokan et al. [38]	x	x						
Gangannagaripalli et al. [26]	x	x				x	x	
Gorchs‐Font et al. [49]								x
Grunwald et al. [57]	x	x				x		
Haunch et al. [31]		x				x		
Ijaz et al. [126]		x						
Law et al. [15]		x	X					
McKenzie et al. [51]								
Miljand et al. [55]	x						x	
Morris et al. [50]								x
Morton et al. [58]		x	X			x		
Osterman et al. [46]	x		X					
Petrova et al. [27]	x	x				x	x	
Price et al. [32]		x				x	x	
Provencher et al. [59]	x	x						
Savelli et al. [42]	x	x	X	x		x		
Siersbaek et al. [39]		x	X			x		
Sims et al. [33]	x	x	X			x		
Singh et al. [60]		x				x		
Tsang et al. [28]								x
VanderKaay et al. [37]		x				x		
Wade et al. [29]								x
Warner et al. [47]	x	x	X			x		
Abrahamson et al. [45]	x	x	X			x		
Bulley et al. [120]	x	x	X			x		
Carroll et al. [43]			X		x	x		
Corey et al. [122]	x	x				x		
Maidment et al. [119]						x		
Stabler et al. [44]	x		X			x	x	
Teeling et al. [118]				x	x	x		
Thijssen et al. [14]	x		X			x	x	
Waldron et al. [121]	x	x	X			x		
Zibrowski et al. [123]	x	x	X			x		

Abbreviation: IPT, initial program theory.

Advisory groups were commonly reported to be involved in clarifying the scope of the review (*n* = 18 realist review; *n* = 7 RRRs). Their role could be to ensure the relevance of the review question; Thijssen et al. [14] reported how the advisory group confirmed that the research question for the review addressed relevant gaps in the literature and covered key interests within the field of practice. Involvement in the development of initial program theories (IPTs) was also often commonly reported (*n* = 23 realist review; *n* = 5 RRRs). For example, Chadborn et al. [16] reported how the advisory group was able to sense‐check the emerging program theories to ensure that they reflected their real‐world experience.

To a lesser extent, advisory groups were reportedly involved in the search strategy stage (*n* = 9 realist reviews; *n* = 7 RRRs), either to serve as a safety check to ensure no relevant literature was missed [15, 16, 42, 43], to identify gray literature [30, 44, 45, 46], to assist in search terminology [45, 47] or to highlight when further searches may be needed [14, 33, 39].

Advisory groups were rarely reported to be involved in the selection and appraisal of evidence (*n* = 1 realist review; *n* = 1 RRR) or data extraction (*n* = 2 RRRs). However, at the later stage of data analysis and synthesis, advisory group involvement was again commonly reported (*n* = 19 realist reviews; *n* = 10 RRRs). While advisory groups were mentioned throughout this stage, notably, they were often reported in later stages of the synthesis and theory validation; for example, to discuss the utility of the emerging theories [17] and to see if the emerging PTs reflected real‐world experience [16, 33, 54]. Haunch et al. [31] described what this looked like in practice: “Refined context‐mechanism‐outcome configurations and examined supporting evidence in three 60–90 min researcher‐led discussions during November–December 2019 with our stakeholder groups which included: residents and relatives (group 1) and managers (group 2) of long‐term care facilities, and our Study Steering Committee (SSC) members (including representatives from provider organizations, policymakers, regulators, methodologists, and members of the public). Stakeholders were invited to discuss and critically comment on the resonance, relevance, and gaps in our theories.” Advisory groups were also involved in dissemination and knowledge translation of findings (*n* = 6 realist review; *n* = 2 RRRs), for example, by suggesting wording [14], feeding into a publication draft [41], or ensuring the findings were accessible to end users [54].

In other reviews, it proved challenging to code advisory group involvement to a specific stage, for example, when terms such as “ongoing consultation” were used [38], or when it was reported that the advisory group informed the study design more generally [41, 46]. Furthermore, five reviews [28, 29, 49, 50, 53] mentioned use of an advisory group but did not provide further details on their involvement.

### 3.5 Terminology

The terminology used to describe advisory groups varied greatly within the reviews. A list of 23 unique terms was found across the realist reviews, with seven further unique terms found across the RRRs. Tables 3 and 4 document these terms. Most commonly, 11 reviews used no clear term, with many using nonspecific language relating to stakeholders, for example, “stakeholder engagement,” “consultation with expert stakeholders,” or “involvement of stakeholders,” and going on to describe their role in advising on the review. However, these studies were included only if they described a group or panel external to the research team used to inform and guide the review and not if they described stakeholders as an individual participant, where for example, interviews were transcribed and coded, this was not included. Four reviews [25, 33, 57, 129] even used different terms within the same paper. While no clear preferred term emerged, some commonalities were seen across the reviews. For example, “advisory group” or versions of this were reported by nine reviews [15, 17, 25, 26, 27, 28, 29, 30, 130], while “stakeholder group” or versions of this were reported by eight reviews [25, 27, 31, 32, 33, 34, 35, 36]. Additionally, variations of “expert panel” were reported by five reviews [37, 38, 39, 40, 41].

**Table 3:** Terminology used to describe advisory groups in realist reviews.

Frequency	Terminology Used in Realist Reviews
**11**	Nonspecific language relating to **stakeholders**
	For example may have used terms such as: stakeholder consultation; consultation with stakeholders; consultation with expert stakeholders; stakeholder discussions; discussions with stakeholders; stakeholder input; stakeholder engagement; engaged with stakeholders; involvement of stakeholders
**9**	**Advisory group** (or version of this for example: “clinical advisory group”; “expert advisory group”; “interdisciplinary advisory group”; “patient advisory group”; “stakeholder advisory group”; “youth advisory group”; “panel of expert advisory group members^a^”)
**8**	**Stakeholder group** (or version of this for example: “key stakeholder group”; “group of stakeholders”; “diverse stakeholder group”)
**5**	**Expert panel** (or version of this for example: “formal expert panel”; “technical expert panel”; “panel of expert advisory group members^a^”)
**2**	**Reference group** or project reference group
**2**	**Steering group**
**1**	**Consultation with nonacademic/academic research partners**
**1**	**Content expert group**
**1**	**Expert review committee**
**1**	**Patient participatory group**
**1**	**Study management group**

*Note*: To note, some used multiple groups with different terminology for each.

^a^ Coded under two headings for expert panel and advisory group.

**Table 4:** Terminology used to describe the advisory group in rapid realist reviews.

Frequency	Terminology used in rapid review
**4**	**Expert panel**
**4**	**Reference Panel** or **Local reference panel or Local reference group**
**2**	Nonspecific language relating to **stakeholders** (e.g., use of terms such as: “key stakeholders”; “stakeholder engagement”)
**1**	**Advisory panel**
**1**	**Core reference group**
**1**	**Expert stakeholder group**
**1**	**Stakeholder team**

*Note*: To note, some used multiple groups with different terminology for each.

For RRRs the terminology of "expert panel" (*n* = 4) and “reference panel/group” (*n* = 4) were reported most frequently. This is in keeping with the suggested terminology in guidance for RRRs Saul et al. [4] However, these suggested terms were used in less than half of the RRRs that used advisory groups, with alternative terms including “core reference group” [122] or “expert stakeholder group” [45].

### 3.6 Use of multiple advisory groups

Multiple groups (*n* = 7 realist reviews; *n* = 4 RRRs) within the same review were also reported. For example, Petrova et al. [27] reported both a patient advisory group and a professional advisory group that met separately. For RRRs, Saul et al. [4] suggested using two groups: a local reference group and an expert panel. This can be seen in practice in the RRR by Waldron et al. [121] who reported an expert panel, with multiple stakeholders, including clinicians and policymakers, along with a separate reference panel of members reflecting international research expertise in the field.

### 3.7 Ethical approval

Most reviews did not report ethical approval. For example Warner et al. [47] noted they did not seek ethical approval given that the advisors had a consultative role throughout the review process and were not study participants. Seven reviews [15, 16, 25, 26, 29, 51, 52] and two RRRs [118, 120] reported seeking ethical approval. However, approval may have targeted other primary data collected separately to an advisory group.

### 3.8 Reflections on the use of advisory group

The majority of reviews did not give details of how the advisory group impacted the study. However, Calderón‐Larrañaga et al. [25] described how an advisory group impacted and strengthened a review. In their review, they specifically highlighted how input from their group expanded their understanding of their topic and allowed for further refinement of literature‐based findings with real‐world experience. Additionally, Price et al. [32] noted that the advisory group contributed significantly to recommendations and the dissemination of their findings.

Some challenges in using an advisory group were also reported. For example, Petrova et al. [27] reported that changes in the membership of the group over time meant that care needed to be taken to bring new members up to speed with the project. A further challenge they noted was that clear expectations had not been set for the groups' role in the later data analysis and synthesis stage. This led to the group providing general opinions, rather than specific input to the PTs. Clear expectations of role should be set for an advisory group from the outset with potential benefits from using established PPI groups with experience depending on the needs of the review.

### 3.9 Reporting on advisory groups

Overall, the level of reporting on advisory groups was poor, with insufficient details of who was involved, for what purpose and at what time points. For some headings on the data extraction form such as “level of involvement,” it was often not possible to extract information based on the minimal details given. For example, reviews mentioned using an advisory group without providing further detail on the extent of their involvement [28, 34, 49, 50]. In contrast, other reviews provided significant levels of detail on the process. One example is Petrova et al. [27] with details reported in the Supporting Information—including reasons for the use of an advisory group, why and how they recruited members, the role of the group and reflections on the impact of the group. This example offered a transparent process for involving an advisory group. By sharing such reflections, future research can learn from insights when deciding when and how to engage an advisory group. Reporting advisory groups contributions, alongside the contributions of other participants, often led to a lack of clarity of who did what. Of those reporting details, the majority reported these within the main text of the peer reviewed publication. Five reviews referenced additional details available in Supporting Information [16, 27, 35, 48, 54] and three referenced more details in a previously published protocol paper [17, 42, 52]. For RRRs, one included further details in Supporting Information [120], one in a protocol paper [45], and one mentioned details in both the Supporting Information and a protocol paper [123]. These were all retrieved and extracted for further information, however varying levels of details were given in these additional files.

### 3.10 Suggested items for reporting of advisory groups in realist reviews

It is important to highlight that if an advisory group is used, it becomes a component of the review methodology. As such, appropriate documentation and reporting of the advisory group should ensure methodological transparency. This study took a best‐fit‐framework synthesis approach using the ACTIVE framework for stakeholder involvement in systematic reviews [9] to inform the study and assist in development of a “Suggested Items for Reporting of Advisory Group in Realist Review” framework. Based on the findings of this study and the original ACTIVE framework [9], and further informed by the TiDier guidance [131] and the Abrams et al. analytical framework [12], the following items in Table 5 may be useful to support reporting advisory group use in a realist review.

**Table 5:** Suggested items for reporting of advisory group in realist reviews based on the ACTIVE framework [9] and further informed by the TiDier guidance [131] and the analytical framework used by Abrams et al. [12].

Suggested items for reporting of advisory group in realist review^a^	
Purpose	What was the purpose of the advisory group for this review?
Who	Who was involved and what is the rationale for their involvement?
Recruitment	How were members recruited?
Mode	Mode of involvement ○ At what timepoints/what frequency were they consulted/did membership of the group change or remain the same throughout? ○ What methods were used? (e.g., meetings, document review, workshops, and were these virtual or in‐person) ○ Did the group meet collectively, as subgroups or individually?
Stage of review	What stage(s) were they involved in? i.e. clarifying scope, developing IPTs, search strategy, selection and appraisal, data extraction, data analysis and synthesis, dissemination
Impact	What was the impact of advisory group involvement? ○ Did the review change direction or scope based on the advice of the advisory group? ○ Any specific actions that were taken based on the advice of the advisory group?
Reflections	Any reflections on the involvement and use of an advisory group in your review?
Terminology	Is the terminology chosen to describe the advisory group within the paper consistent throughout?

^a^ If multiple groups are used, consider completing the above for each group.

### 3.11 Best‐fit framework synthesis: Suggested items for reporting of advisory groups in realist reviews

The headings relating to “who,” “recruitment,” “mode,” and “stage” in Table 5 were all based on the ACTIVE framework [9], however the prompts relating to these headings were adapted. For example in the “mode of involvement” heading in the ACTIVE framework [9], under “methods” they asked whether the interaction was direct or nondirect, alternatively, we used the prompts “what methods were used? (e.g., meetings, document review, workshops, and were these virtual or in‐person)” and “did the group meet collectively, as subgroups or individually?” as we felt more useful information may be given. For the heading of “recruitment,” the ACTIVE framework [9] gave a number of options for example “open/closed” or “fixed/flexible” with an explanation on a following page as to what each category meant, however in the adapted framework we left the prompt open for this heading with “how were members recruited?”, which may be more user friendly, allowing for a brief narrative explanation of the recruitment process. For the ACTIVE framework [9] heading of “level of involvement,” we found this to be quite subjective to code when analyzing the papers, and not as useful as the new heading of “purpose” which prompts the researcher to document what the intended purpose of the advisory group was. Similarly, the new heading of “impact,” which was present in the Abrams et al. [12] analytical framework, allows for documentation of any changes to the review that were made based on advisory group inputs. For the “stages” heading, the stages of the review were adapted from systematic reviews to those found in realist reviews.

Further to this we added two more headings for “reflection” and “terminology.” We believed that documenting reflections could give future reviews useful information when planning their advisory groups, and given the ambiguity found in terminology we felt it was important to include this prompt to ensure what language is used is consistent throughout. Notably, there was a lack of clear terminology in describing advisory groups across the reviews. It is recognized that different terminology may make sense to each review. For example, where individuals donate time to advise based on their experience but are not a direct beneficiary themselves, the term “advisory group” may make sense, whereas reviews where multiple members will be end users of the output of the review, terms related to a “stakeholder group” may feel more appropriate. Potentially the group itself could decide what term best represents their intended contribution. What is most important is ensuring that the purpose and use of the advisory group is clear. Following this, whatever terminology is chosen should remain clear and consistent throughout the study and reporting.

### 3.12 Considerations when planning use of an advisory group

It is important to note that in standard realist reviews, advisory groups are not a requirement and were used in less than half of the reviews identified. However, aligned to guidance from Saul et al. [4] advisory groups are common practice in RRRs. There were no major differences in how advisory groups were used in realist reviews and RRRs, with the exception that RRRs appeared more likely to form two groups than a realist review. However, given the small sample size of RRRs this finding should be taken with this caution. It may be helpful for investigators to examine whether an advisory group can add value to the review and, if so, in what way, before choosing whether or not to use one. Some topics may lend themselves to use of an advisory group, for example, where information or evidence is less plentiful; or where multiple evidence or options make it challenging to navigate, an advisory group can assist in prioritizing focus areas based on importance to end users of the review. Abrams et al. [12] used an analytical framework with questions posed to guide their analysis of PPI in realist reviews; we have adapted and further expanded upon the suggested prompts in their blog [20] to encompass advisory groups. For example, we have added a prompt on ethical considerations, such as how the group's inputs will be used and considering the policy of the local ethics board. Additionally, we have added a prompt to consider what terminology would be most appropriate to describe the advisory group based on the purpose or intention of the realist review. Therefore we propose the following considerations (see Table 6), which may support investigators in deciding on and planning the use of an advisory group in a realist review.

**Table 6:** Considerations for deciding on and planning use of an advisory group in a realist review, informed by the ACTIVE framework [9] and expanding upon the reflective prompts suggested by Abrams et al. [20].

Considerations for deciding on and planning use of an advisory group in a realist review
• What is the purpose of an advisory group for this review?
i.e., Why would we choose to use an advisory group?
What are the needs of the review?
What impact do we hope the advisory group may have?
• Who will be involved as members of the advisory group?
i.e., What experiences could assist in informing and advising this review?
Who would have that experience?
How will we recruit members? (e.g. existing groups, open call, etc.)
• What will the advisory group members be asked to do?
i.e., What will their role be?
How will we set clear expectations with the advisory group members on their roles?
• How will we engage with the advisory group?
i.e., What mode of interaction (meetings, emails, workshops, etc.) will be used?
Will the group meet as one group, subgroups, or individually?
Will we meet the group virtually or in person?
• What commitments are we asking for?
i.e., What is the time commitment, including how frequently will they meet?
Are there any expectations to complete workload between meetings?
• Will members be acknowledged for their time?
i.e., Are any compensation, remuneration or reimbursements required?
Will members be formally acknowledged in any dissemination, or be considered for author contributions if they meet the criteria?
• Do I need to consider getting ethical approval for this advisory group?
i.e., How do we plan on using the group's inputs?
Would any direct quotes from the group be used?
What is the policy of our local ethics board?
• Is the advisory group being used to fulfill funder (or other agency) requirements for public and patient involvement (PPI)? If so, how can the PPI members involvement and impact be recorded and reported during and after the review?
• How will we feed back to the advisory group on the review (both during and upon completion of the review)?
• How will we decide what contributions of the advisory group to take forward and not take forward?
• What terminology is most appropriate to describe this advisory group?

^a^ If ethical approval is needed, consider whether the group is an advisory group or if they are a participant/focus group instead.

## 4 DISCUSSION

In a realist review, an advisory group was most frequently found to be constituted of members external to the research team who could provide external and independent perspectives to inform the review based on their experience of the topic area. Advisory group members tend to be selected to capture varied experiences, from lived and professional experiences to policy and research expertise in the field of interest. To a lesser extent, members may be selected for their methodological knowledge to inform and guide the review process when the review team feels a need for support. Advisory groups can be involved in one or more stages of the review. Members most commonly meet collectively as a group with the research team, or in subgroups, however they may also meet with the research team individually. The role of the advisory group differs to that of the research team, such that they consult, advise and inform the review rather than carry out the review process itself. Additionally, as their role is to consult and advise, they differ from a research participant who is recruited to provide data for a study, and consequently, ethical approval is rarely sought. While advisory group members tend to be external to the review team, there may be instances, where due to their level of involvement, some members may over time become members of the review team and authors on subsequent publications. Where this does occur it should be documented for transparency.

Experientially, and as seen in the literature, realist reviewers are involving groups or panels external to the research team who provide external and independent perspectives informing their reviews. Advisory groups provide a specific vehicle for involvement, moving beyond the diffuse nature of stakeholder involvement at large, to question and shape research that is appropriate and relevant [132, 133]. Research advisory groups are considered an effective way to involve the public [134], yet the findings of this article demonstrate the wide range of use and reporting of advisory groups in realist reviews and RRRs. They may have different implications and purposes which have not yet been captured. This paper fills a gap by beginning to put parameters down to understand and distinguish the ambiguity between using these different types of groups and stakeholders. Various terminology is used to describe the individuals or stakeholders external to a review team who are involved in supporting or informing the review. When terminology is used interchangeably or in combination with little distinction between them, this can cause confusion in practice [135]. This is reflected in a similar challenge when involving stakeholders or PPI in research and clarifying the boundaries between their involvement as research participants versus PPI contributors who take on an active role in the research [136]. It is challenging to implement a methodological step, such as involving advisory groups, when there is such a range in terminology used and different implications or definitions. Notably, no set terminology is used to describe an advisory group, and we suggest that terms can be selected based on what makes the most sense to the group once clarity of the group's parameters in the context of the study are transparently reported.

Furthermore, advisory groups are used in different ways and to different extents. Similar to Abrams et al. [12] we found that reporting of the details and level of involvement of advisory groups is often poor. This makes it difficult to understand how the advisory group contributed to or influenced the realist review, while also posing challenges for those looking for guidance on how to involve an advisory group. While reporting guidelines and quality standards exist for a range of evidence reviews, adherence to these guidelines is variable [137, 138]. Some items are more straightforward and easier to report sufficiently [138]. The RAMESES publication standards for realist review aim to provide a resource to improve the reporting of realist reviews by providing explanations and examples of how each item in the standards has been reported in the literature [5]. Providing detailed examples and explanations of how researchers have addressed these steps of engaging an advisory group can contribute to more comprehensive and transparent reporting, therefore informing evidence‐based decision‐making [139]. Similarly, the Guidance for Reporting Involvement of Patients and the Public (GRIPP) checklist was developed to provide guidance for reporting PPI in health and social care research [140]. While adherence to reporting GRIPP and GRIPP2 vary, encouraging requirements from journals and funding organizations can help to improve comprehensive and quality reporting [141]. Initiatives such as the EQUATOR network promote the availability and standardized use of transparent and accurate reporting guidelines to enhance the value and reliability of published research [142].

Staniszewska et al. [143] emphasize how inadequate reporting makes it challenging to interpret and synthesize findings across studies, further fragmenting the evidence base and researchers' ability to translate lessons learned. This remains the case for reporting how advisory groups are involved, influencing future researchers' understanding of the methodological process and therefore their ability to apply it in their own work. For this study, a “best‐fit” framework synthesis used the well‐tested and developed ACTIVE framework [9] to analyze the context of advisory groups in realist reviews, to add granularity and nuance i.e. the detailed data about our phenomenon of interest to the existing framework. The “best‐fit” framework synthesis is recognized as a valuable method that provides an explicit and transparent approach to supplementing or complementing an existing theoretical framework with the addition of new concepts. To support future realist researchers, the prompts in Table 6 present considerations that can be reflected upon in the planning stage of the review and Table 5 exhibits suggested items for reporting that can be used when disseminating their study. While the majority of the elements of the Abrams et al. [12] analytical framework align with the elements of the ACTIVE framework [9] (e.g. who was involved and how), their addition of reporting the impact of the advisory group was also included.

## 5 LIMITATIONS AND DIRECTIONS FOR FUTURE RESEARCH

This study identified a sample of reviews from a 1‐year period and the extracted data was limited by the level of detail reported and available in the literature. An additional limitation is that this study did not explore specific topic areas or target populations for each review. This potentially may have identified differences between advisory groups based on the review topic or population. This could be explored in future studies on advisory groups in realist reviews. This review of the literature provides the first step towards developing formal reporting guidelines on the use of advisory groups in realist reviews. Building off the ACTIVE framework [9], as well as incorporating the established TiDier guidance [131], and with learning from Abrams et al. [12, 20] a checklist which would enable the comprehensive and transparent reporting of using advisory groups in realist reviews and RRRs could be further developed. This article serves as an initial step in developing more formal guidelines which can be a supportive tool for reporting, building off of the RAMESES realist synthesis publication standards [5]. As outlined by Moher et al. [144] these findings would need to be taken forward for stakeholder consultation and the development of formal reporting guidelines. Therefore, the above items for reporting serve as suggestions for those completing realist reviews rather than formal guidelines.

## 6 CONCLUSIONS

Advisory groups can be a useful addition to a realist review, where members external to the research team provide external and independent perspectives to inform the review based on their experience of the topic area. The group can be involved at any stage of the review. The purpose of the advisory group can be identified depending on the review topic and the groups' use guided by the needs of the review. Clear documentation can assist in the transparency of the use of an advisory group. This study provides clarity on what an advisory group is and suggests items for reporting their use in a realist review.

## AUTHOR CONTRIBUTIONS

**Jessica Power**: Conceptualization; data curation; formal analysis; investigation; methodology; project administration; validation; writing—original draft; writing—review and editing. **Sara Dada**: Conceptualization; data curation; formal analysis; investigation; methodology; validation; writing—review and editing. **Andrew Booth**: Conceptualization; methodology; writing—review and editing. **Aoife De Brún**: Conceptualization; methodology; writing—review and editing. **Brynne Gilmore**: Conceptualization; formal analysis; investigation; methodology; validation; writing—review and editing.

## CONFLICT OF INTEREST STATEMENT

The authors declare no conflict of interest.

## PEER REVIEW

The peer review history for this article is available at https://www.webofscience.com/api/gateway/wos/peer-review/10.1002/cesm.12073.

## Supporting information

Supporting information.

Supporting information.

Supporting information.

## Data Availability

The data that support the findings of this study are available from the corresponding author upon request.
