# Supplementary material for: Advisory groups in realist reviews: Systematically mapping current research and recommendations for practice
Source: Cochrane Evid Synth Methods. 2024 Jun 11;2(6):e12073. doi: 10.1002/cesm.12073 (PMC11795975; doi:10.1002/cesm.12073)
Supplement: Supplementary file 1 — Supporting information. [file CESM-2-e12073-s002.docx]

**Supplemental File 1. Search for best fit framework**

**Known citations used for backward and forward citation searching**

| Known Citation | Ref list search | Forward Citation (google scholar) | Date Searched |
| --- | --- | --- | --- |
| Shippee, N.D., et al., Patient and service user engagement in research: a systematic review and synthesized framework. Health Expect, 2015. **18**(5): p. 1151-66. | 111 | 503 | 27/09/22 |
| Pollock, A., et al., Development of the ACTIVE framework to describe stakeholder involvement in systematic reviews. Journal of health services research & policy, 2019. **24**(4): p. 245-255. | 60 | 43 | 27/09/22 |
| Haddaway, N.R., et al., A framework for stakeholder engagement during systematic reviews and maps in environmental management. Environmental evidence, 2017. **6**: p. 1-14. | 35 | 79 | 27/09/22 |
| Abrahamson, V., et al., *The role (or not) of patients and the public in realist reviews*. 2020: Accessed from: <https://www.spcr.nihr.ac.uk/news/blog/the-role-or-not-of-patients-and-the-public-in-realist-reviews> on 27th Sept 2022 | 7 | 2 | 27/09/22 |
| Feldmann, J., M.A. Puhan, and M. Mütsch, Characteristics of stakeholder involvement in systematic and rapid reviews: a methodological review in the area of health services research. BMJ Open, 2019. **9**(8): p. e024587. | 173 | 5 | 27/09/22 |
| Total for: Title / Title+Abstract Screening | 386 | 632 | Total n=1018  (includes duplicates) |

**From above search: eight frameworks identified and brought forward for discussion**

- Ball, S., et al., Patient and public involvement in research: Enabling meaningful contributions. 2019, Santa Monica, CA: RAND Corporation.
- Haddaway, N.R., et al., A framework for stakeholder engagement during systematic reviews and maps in environmental management. Environmental evidence, 2017. **6**: p. 1-14.
- Keown, K., D. Van Eerd, and E. Irvin, Stakeholder engagement opportunities in systematic reviews: knowledge transfer for policy and practice. J Contin Educ Health Prof, 2008. **28**(2): p. 67-72.
- Kreis, J., et al., Consumer involvement in systematic reviews of comparative effectiveness research. Health Expectations, 2013. **16**(4): p. 323-337.
- Pollock, D., et al., Moving from consultation to co-creation with knowledge users in scoping reviews: guidance from the JBI Scoping Review Methodology Group. 2022(2689-8381)
- Pollock, A., et al., Development of the ACTIVE framework to describe stakeholder involvement in systematic reviews. Journal of health services research & policy, 2019. **24**(4): p. 245-255.
- Shippee, N.D., et al., Patient and service user engagement in research: a systematic review and synthesized framework. Health Expect, 2015. **18**(5): p. 1151-66.
- Staniszewska, S., et al., Developing a Framework for Public Involvement in Mathematical and Economic Modelling: Bringing New Dynamism to Vaccination Policy Recommendations. Patient, 2021. **14**(4): p. 435-445.

**Best fit framework selected as starting point**

- Pollock, A., et al., Development of the ACTIVE framework to describe stakeholder involvement in systematic reviews. Journal of health services research & policy, 2019. **24**(4): p. 245-255.
