# Supplementary material for: Advisory groups in realist reviews: Systematically mapping current research and recommendations for practice
Source: Cochrane Evid Synth Methods. 2024 Jun 11;2(6):e12073. doi: 10.1002/cesm.12073 (PMC11795975; doi:10.1002/cesm.12073)
Supplement: Supplementary file 2 — Supporting information. [file CESM-2-e12073-s003.docx]

**Supplemental File 2. Search strategy – search for realist reviews 2021**

Table 1. Search strategy for search carried out on all databases 6^th^ Sept 2022 by JP, limit of 2021 applied to all. Results exported to covidence

| **Database** | **Host** | **Search Terms** | **Results** |
| --- | --- | --- | --- |
| Cinahl Complete | EBSCO | TI (“realist systematic review*” or “realist review*” or “realist synthes*”) OR AB (“realist systematic review*” or “realist review*” or “realist synthes*”)  Limit: Dates 2021 only | 66 |
| Cochrane | Cochrane library | ("realist systematic review*" or "realist review*" or "realist synthes*"):ti or ("realist systematic review*" or "realist review*" or "realist synthes*"):ab  Limit: Dates 2021 only | 0 |
| Embase | Ovid | ('realist systematic review*':ti OR 'realist review*':ti OR 'realist synthes*':ti OR 'realist systematic review*':ab OR 'realist review*':ab OR 'realist synthes*':ab) AND [2021-2021]/py  Limit: Dates 2021 only | 140 |
| ERIC | EBSCO | TI (“realist systematic review*” or “realist review*” or “realist synthes*”) OR AB (“realist systematic review*” or “realist review*” or “realist synthes*”)  Limit: Dates 2021 only | 2 |
| Medline | EBSCO | TI (“realist systematic review*” or “realist review*” or “realist synthes*”)  OR AB (“realist systematic review*” or “realist review*” or “realist synthes*”)  Limit: Dates 2021 only | 144 |
| Psycinfo | EBSCO | TI (“realist systematic review*” or “realist review*” or “realist synthes*”)  OR AB (“realist systematic review*” or “realist review*” or “realist synthes*”)  Limit: Dates 2021 only | 28 |
| Social Services Abstracts | ProQuest | ti("realist systematic review*" OR "realist review*" OR "realist synthes*") OR ab("realist systematic review*" OR "realist review*" OR "realist synthes*")  Limit: Dates 2021 only | 3 |
| Sociological Abstracts | ProQuest | ti("realist systematic review*" OR "realist review*" OR "realist synthes*") OR ab("realist systematic review*" OR "realist review*" OR "realist synthes*")  Limit: Dates 2021 only | 5 |
| Web of Science Core Collection | Clarivate Analytics | (TI=("realist systematic review*" OR "realist review*" OR "realist synthes*")) OR AB=("realist systematic review*" OR "realist review*" OR "realist synthes*")  Limit: Dates 2021 only | 149 |
|  |  | Total | 537 |
|  |  | Total after removal of duplicates (duplicates n=339) | 198 |
