# Supplementary material for: Advisory groups in realist reviews: Systematically mapping current research and recommendations for practice
Source: Cochrane Evid Synth Methods. 2024 Jun 11;2(6):e12073. doi: 10.1002/cesm.12073 (PMC11795975; doi:10.1002/cesm.12073)
Supplement: Supplementary file 3 — Supporting information. [file CESM-2-e12073-s001.docx]

**Supplemental File 3. Copy of data extraction template**

**Screen Full Text**

**Screen the full text of paper. If it is not a published realist review --> Click 'move study to full text review.' If it is a realist review, continue extraction.**

**Demographics**

**Author**

**Year**

**Country of lead author**

**Country or region of review focus**

**Review topic**

(e.g. health, education, etc)

**Rapid Review**

1. Yes
2. No

**Advisory Group Filter Questions**

**Did the review report the involvement of participants/stakeholders that were external to the review team?**

Note: When reading study if unclear if advisory group, tag as "unclear" for double extraction

1. Yes, advisory group used -> Continue extracting
2. Yes, participants used, but no advisory group -> Can stop extraction here
3. No participants reported -> Can stop extraction here
4. Other

**If yes, what language did they use for the advisory group? (e.g. expert group, steering committee)**

**Does this paper warrant further extraction?**

**Did they report any details on the advisory group (i.e. who, why or what they did)?**

1. Yes -> Continue extraction
2. No i.e. mentioned an advisory or expert group used but gave no further details -> can stop extraction here)

**Continue for Studies that gave details:**

**How were the details of the advisory group reported?**

1. Main review paper
2. Referenced Protocol paper
3. Referenced Supplemental file
4. Other

**If they referenced a protocol or other paper with information about their advisory groups please provide link/doi here:**

If yes, use its information as appropriate in the rest of the extraction

**Purpose of Advisory Group**

**Did the paper give a definition or justification for use of advisory group? If yes, what did they say**

**If they gave a definition or justification did they reference another paper/guidance?**

This question just to add the reference if they gave one

**What did the Advisory Group Do?**

**At what stage did involvement occur?**

|  | **Example of what they did** |
| --- | --- |
| **Clarifying Scope** |  |
| **IPT Development** |  |
| **Search Strategy** |  |
| **Selection & Appraisal of Documents** |  |
| **Data Extraction** |  |
| **Data Analysis and Synthesis** |  |
| **Theory validation (following synthesis)** |  |
| **Dissemination (knowledge translation)** |  |
| **Mutiple stages reported together / Does not fit one specific stage** |  |
| **Did not report / Unclear** |  |

**Did they report any specific impact or changes based on advisory group input?**

For example was the outcome of the review impacted by the involvement of the advisory group

**Did they reflect on the process of using an advisory group?**

**Level of involvement**

**Level of Involvement of advisory group - If applicable please give examples below**

Leading: “Initiating the review; lead responsibility for carrying out and completion of review - Tasks will include authorship of a review, and may include any activities associated with review completion, including key decisions relating to the methods and execution of the review.”

Controlling: “Working in partnership with researchers, with varying degrees of control or influence over the review process. Making decisions and/or controlling one or more aspects of the review process, in collaboration with or under the guidance of the review authors - Tasks may include defining outcomes of interest, inclusion criteria, key messages arising from review findings and writing a plain language summary. In completing tasks people have control over final decisions, such as application of inclusion criteria, categorisation of interventions, or recommendations for clinical practice”

Influencing: “Stating, commenting, advising, ranking, voting, prioritising, reaching consensus. Providing data or information which should directly influence the review process, but without direct control over decisions or aspects of the review process. - Tasks may include assisting with review tasks, such as hand-searching, screening, data extraction and assessment of risk of bias, possibly in a co-reviewer role. Tasks may include peer review, such as commenting on a protocol, systematic review or plain language summary.”

Contributing: “Providing views, thoughts, feedback, opinions or experiences. Providing data or information which may indirectly influence the review process. People may be participants in a research study (e.g. focus groups or interviews). - Tasks may include sharing views or opinions, for example within a focus group of interview. May include ranking, voting or prioritising as participants in a research study (e.g. Delphi study).”

Receiving: “Receiving information about the systematic review, or results of the review - Tasks may include attending events, or reading or listening to information about the review. While the results of a review may be discussed, these discussions do not influence the review process in any way.”

|  | **Example** |
| --- | --- |
| **Leading** |  |
| **Controlling** |  |
| **Influencing** |  |
| **Contributing** |  |
| **Recieving** |  |
| **Not enough information / Unclear** |  |

**Mode of Involvement**

**Did they report how many times they engaged with the advisory group? If so how many?**

**Were the advisory group involved as**

1. One-time involvement (at specific stages of review)
2. Continuous involvement (throughout review)
3. Periodic (e.g. at set times points, for specific activities, but not involved continuously at every stage)
4. Not reported
5. Other

**Methods of Involvement**

**What did the interaction between the group and review team look like? (e.g. meetings, workshops etc)**

Open text to describe. Only code questions below if obvious

**What did the interaction between the group and review team look like?**

Was there direct (e.g. face-to-face or online meetings) or indirect interactions?

In the indirect method, involvement occurred through participation in an electronic Delphi method to reach group consensus on a particular issue relating to the review. Where both direct and indirect methods were used in the same systematic review we categorised this as ‘direct’, since there was direct interaction in addition to other approaches.

1. Direct
2. Indirect
3. Did not Say
4. Other

**Who was part of Group and how Recruited**

**Who was involved in the advisory group?**

Please specify…. (e.g. service users, service providers, topic experts, gender, number etc)

**Did they explain how or why they recruited this advisory group?**

Can be broad, what methods they used to recruit, why they picked who they did, can copy/paste, and can code below only if its obvious

**Did the study use participants in addition to the advisory group? If so please state who (e.g. patients) and what they contributed to the study (e.g. theory refinement)**

**Coding for Recruitment (only complete if clear, otherwise skip to miscellaneous questions below and open boxes in section above can be enough for now)**

**How was the advisory group recruited?**

“Open’ recruitment refers to providing opportunities for involvement through advertisement to the general population, allowing anyone to volunteer to get involved.” e.g. twitter broadcast.

“Closed recruitment strategies focus on inviting only specific people to participate. Closed strategies include invitation of known individuals or recognised experts, recruitment from membership of an existing group, or purposive sampling to achieve representation of people with key pre-determined characteristics, experience or expertise”

1. Open
2. Closed
3. Did not mention
4. Other

**If open recruitment, was the group**

Use 'other' to provide any further description/explanation

1. Fixed (same group throughout)
2. Flexible (different people contributed different times, recruitment changed as progressed)
3. No mention
4. Other

**If closed/targeted recruitment, was it**

Invitation to specific known individuals

Existing group e.g. PPI group for diabetes already set up asked to join

Purposive sampling - targeted effort made to include people from specific groups/knowledge areas

1. Invitation
2. An existing group
3. Purposive sampling
4. No mention
5. Other

**Miscellaneous Questions**

**Was any data recorded/transcribed/quotes used from the advisory group?**

1. Yes
2. No
3. Unclear
4. Other

**Ethics and Funding**

**Was ethical approval sought?**

If yes please give details of ethical approval (where from or ref no) in the other box

1. Yes
2. No
3. Other

**If ethical approval was sought, did they specifically mention this was for an advisory group? Give quote/details if so.**

**If provided, who was the funder?**

**Notes**

Any other notes
